# Heavy Foils

**Published:** 1870-04

**Authors:** Henry S. Chase


### SELECTED ARTICLES.

### ARTICLE V.

### *Heavy Foils.*

By Henry S. Chase, M. D., D. D. S.

The number of dentists who use the heavy foils is rapidly increasing. Dr. Atkinson, of New York, deserves the thanks of the profession for bringing them again to its notice.

Dr. Arthur, of Philadelphia, many years ago advocated the use of Nos. 10 to 15. That was before mallet pressure was known. Foils as heavy as 30, 50 and 120 could not easily be manipulated by hand pressure, and one cannot go much higher than Nos. 10 or 15, even with a single thickness of a leaf, by hand pressure alone.

When new fashions are introduced many persons go to an extreme which is ridiculous; and so in the use of the heavy gold there is danger of going to an extreme which is neither desirable nor useful, but on the contrary injurious.

Undoubtedly two pure gold plates of No. 20 of the guage could be welded together by serrated instruments and sufficient mallet force.

Now the thickness of our gold foils must be adapted to the strength and condition of the tooth which we are to plug.

A certain amount of mallet force will cause pericemental inflammatiou in one tooth, which would be perfectly indifferent to another tooth. The enamel which would remain intact from any number of blows of a definite weight, from the mallet, in impacting a gold plug, would crack in one or more places by *increase* in the *weight* of blows, given to the gold alone. We have all seen cracks occur in the enamel from concussion produced by blows from the mallet.

The force which is given by a mallet to a plugger, placed on the mass of a gold plug, is diffused throughout the whole substance of the plug, and also every portion of the crown and root, and even to the maxilla itself. Now this concussion produces cracks in the enamel which are injurious to the integrity and durability of the tooth. Therefore the blow should be brought down to that degree of weight which the dentine is able to bear with impunity.

The smaller the mass of gold under the plugger the less force is required for welding. Therefore, the smaller the point of the plugger the lighter may be the blow to produce the desired effect.

To ensure the welding of 120 gold foil, without *too great concussion*, we must use pluggers with very small points. Those measuring No. 20 of the guage plate are as *large* as should be used for No. 120 foil. Smaller ones than these must be more generally used, especially in those places where great strength of plug is required, and the greatest perfection of welding desirable.

For the *margins* of *approximal* cavities, I prefer No. 30 foil, and also for delicate margins on the crowns of bicuspids. The approximal cavities in the incisors, I think, are also easier plugged with Nos. 20 or 30, than with 60 or 120. The undercuts that must be plugged by hand pressure, are also best filled with No. 20.

Nos. 60 and 120 are best manipulated by being cut into small squares, not so wide as the diameter of the cavity ? or by oblong squares, three times as long as wide. They may be consolidated best, by laying them flat and using one or two thicknesses at a time, for impaction. In moderately large cavities, having heavy walls, they may be placed flatways, or edgeways, indifferently. Against the perpendicular walls, it is best to let a small portion of the flat side lie, and with a foot-shaped instrument impact it against the wall at an acute angle.

Nos. 20 or 30 can be used in most cavities, without reference to the position of the strips. I cut these numbers into strips, from a half inch to an inch in length, and from one-sixteenth to one-eighth of an inch wide. But a more even surface can be kept by *folding* any of the heavy numbers upon themselves flatways.

When the surface of the cavity is nearly approached, a more satisfactory plug will be made by always laying the strips flat. Minute depressions are thus avoided in the face of the plug when it is polished.

Have I abandoned No. 2 gold foil? No. There is hardly a day that I do not use it in connection with the heavy foils. I use No. 2 in situations where *strength* of plug is not required, and when density is not desirable. Strength would not be required in the bottom of a *simple* crown cavity, even if very large. Density is not desirable next to a nearly exposed pulp. A plug of a given size will weigh more if made of No. 60 gold, than of No. 2. The greatest possible solidity in a plug is not *always* desirable. The nearer we can approach to the density of the dentine itself, and at the same time, ensure strength and the exclusion of fluids, the better. —*Missouri Dental Journal.*

---

